# The Climatology of Hæmoptosis

**Published:** 1890-01

**Authors:** 


THE CLIMATOLOGY OF HÆMOPTOSIS.—Dr. Roland G. Curtin, of Philadelphia, in an address before the American Climatological Association, June 25, 1889, tabulates the influence of climate on hæmoptosis under two heads: first, the preventive and curative, and, second, the causative:

1. Preventive and curative elements.

*Rarefied air* arrests the ulceration or other diseased processes and lowers the arterial tension. This greatly overbalances the unfavorable tendency of increased heart action and loss of support to the lungs from diminished air-pressure.

*Cold air* contracts the tissues and blood-vessels, thus preventing a flow of blood when such tendency exists. Its general invigorating effects are beneficial.

*Dry air* desicates the pulmonary tissues, decreases the fluidity of the blood, and blocks up the blood-vessels—all favoring the arrest and prevention of bleeding.

*Aseptic air* favors repair and cure of lung disease, and kills or dwarfs the action of the disease-germ.

*Outdoor life*, when not associated with too much exposure, exertion, or fatigue, is beneficial.

*Sunshine* improves the general nutrition.

2. Causative elements.

*Sea-level air*, by its greater density, diminishes the tendency to hæmoptysis, but the increased arterial tension and the moisture usually present in such localities more than counterbalances the beneficial effect of the support given by the air-pressure.

*Salt air* hastens the breaking-down process in tubercular lung disease. The effect is probably good in syphilitic lung troubles, and sometimes in simple chronic inflammatory non-tuberculous lung affections.

*Moist air* hastens the ulcerative process, liquefies the blood and secretions, and renders the tendency to the oozing and flowing of the blood more liable.

*Warm air* relaxes the tissues and blood-vessels and enervates and relaxes the system at large.

Thus he concludes that each ease should be carefully studied in all its phases before deciding upon a change of residence. On a high mountain (say from 5,000 to 10,000 feet [1550–3100 metres]), a residence far removed from the sea-coast, is best for a patient with a tendency to hæmoptysis. At a location of this kind one would probably have not only a rarefied, but also a cold, dry, aseptic air—factors which would be most benficial. Care should be taken that the elevation of the patient should be gradual and not too rapid; otherwise the early effects of a sudden elevation might be followed by unpleasant results. A case of syphilitic phthisis will probably be benefited by sea-air, while a tubercular patient would be injured by it.—*Satellite.*

